# Variations in Protein Concentration and Nitrogen Sources in Different Positions of Grain in Wheat

**DOI:** 10.3389/fpls.2016.00942

**Published:** 2016-06-28

**Authors:** Xiangnan Li, Longjing Zhou, Fulai Liu, Qin Zhou, Jian Cai, Xiao Wang, Tingbo Dai, Weixing Cao, Dong Jiang

**Affiliations:** ^1^National Engineering and Technology Center for Information Agriculture/Key Laboratory of Crop Physiology and Ecology in Southern China, Ministry of Agriculture, Nanjing Agricultural UniversityNanjing, China; ^2^Department of Plant and Environmental Sciences, Faculty of Science, University of CopenhagenTaastrup, Denmark

**Keywords:** protein, pearling, wheat, nitrogen, isotope

## Abstract

The distribution patterns of total protein and protein components in different layers of wheat grain were investigated using the pearling technique, and the sources of different protein components and pearling fractions were identified using ^15^N isotope tracing methods. It was found that N absorbed from jointing to anthesis (JA) and remobilized to the grain after anthesis was the principal source of grain N, especially in the outer layer. For albumin and globulin, the amount of N absorbed during different stages all showed a decreasing trend from the surface layer to the center part. Whereas, for globulin and glutenin, the N absorbed after anthesis accounted for the main part indicating that for storage protein, the utilization of N assimilated after anthesis is greater than that of the stored N assimilated before anthesis. It is concluded that manipulation of the N application rate during different growth stages could be an effective approach to modulate the distribution of protein fractions in pearled grains for specific end-uses.

## Introduction

The grain protein concentration, which to a great extent determines the quality of pasta and bread, is one of the major pricing factors for wheat trading, and is an important nutritional factor for human health (Masclaux-Daubresse et al., 2008). The protein concentration is closely related to the nitrogen (N) content and dry mass in wheat grains and can be separated into the fractions of albumin, gliadin, globulin, and glutenin according to its solubility in different solvents (Malik et al., 2013). Grain N is derived from N that is absorbed after anthesis and from remobilized N that is stored in vegetative organs assimilated before anthesis. Both N sources support the synthesis of the storage proteins in grain (Dupont and Altenbach, 2003), and the former contributes approximately 60–95% to the grain N (Palta and Fillery, 1995). On the contrary, approximately 90% of carbon in grain is acquired by the concurrent photo-assimilates after anthesis (Zhang et al., 2011). In an extreme case, when plants are grown without N supply after the emergence of flag leaf, the remobilization of N from leaves, glumes, stems, and roots contributed 40, 23, 23, and 16% to grain N, respectively (Simpson et al., 1983).

In many studies, the contribution of the pre-anthesis assimilated N to grain N was calculated by the difference in the N stored in vegetative organs at anthesis and maturity (Arduini et al., 2006; Ercoli et al., 2008). Similarly, the contribution of post-anthesis absorbed N to grain N was calculated by the difference between the N in the mature grain and the remobilized N assimilated pre-anthesis (Shi et al., 2012a). However, errors might exist using this method, due to the loss of senescence organs (especially for leaves, which present the highest N concentration and account for a large share of the remobilized N), nutrient leaches, and the fact that the post-anthesis absorbed N might be retained in vegetative organs such as sheathes and stems, which usually depends on the N supply level of the soil and the N fertilization level (Shi et al., 2012a). To overcome these problems, the ^15^N isotope tracing technique is used to discriminate the N that is absorbed at different growth stages and between the concurrent absorbed N and the remobilized N (Kichey et al., 2007). It has been shown that the contribution of N assimilated before anthesis to grain N is largely dependent on the genotypes, soil N status, field management practices such as fertilization and irrigation, and environmental conditions (Ferrise et al., 2010). However, the contributions of N that is absorbed during different growing periods and the N that is remobilized from different vegetative organs to the mature grains have not been well-quantified.

Pearling (debranning) is a new milling technique in the milling and baking industry, where the bran layers are removed sequentially by friction and abrasion operations with a type of device that is a modified rice polisher (Beta et al., 2005). The advantages of pearling before roller milling include improving the semolina yield and the quality of the durum wheat (Dexter and Wood, 1996), enhancing the bread-baking quality (Mousia et al., 2004), depressing the xylanase activity in both whole meal and flour (Gys et al., 2004), and decreasing the α-amylase activity in the flour of pre-harvest sprouted grains of wheat (Hareland, 2003). At the same time, pearling provides us an effective way to investigate the distribution patterns of both the chemical components and quality of flour that are located at different positions in wheat grain, which is essential for separating the milling fractions for specific products (Liu et al., 2007). The distribution patterns of ash, iron, and zinc and the phenolic compounds, with antioxidant activity, have been illustrated in different pearling fractions (Fares et al., 1996; Beta et al., 2005; Liu et al., 2007, 2008). It has also been reported that the flour obtained from pearled wheat was significantly different from the unpearled wheat in terms of flour particle size distribution, percentage of damaged starch granules, starch gelatinization temperature, moisture, and concentrations in the chemical compositions, such as ash and protein (Mousia et al., 2004). In addition, a clear gradient in the protein concentration has been found across the starchy endosperm by microscopy, which was higher in the sub-aleurone cells and lower in the central endosperm cells (Tosi et al., 2011). Moreover, the concentrations of the protein components, such as the gluten proteins, were reported to be expressed in gradient along the different parts of the endosperm (Tosi et al., 2009). However, little is known about the concentration gradient of proteins and protein fractions in different layers of wheat grain and the N sources of those proteins. In this study, the distribution patterns and concentration gradient of the protein and protein components in wheat grain were investigated using the pearling technique, and the ^15^N isotope tracing technique was used to discriminate the N sources of different protein components in different pearling fractions of grain. The results will be essential in understanding the mechanisms of grain quality formation and exploring better N fertilization protocols for super quality wheat production.

## Materials and methods

### Experimental design and materials

Seeds of winter wheat (*Triticum aestivum* L., cv. Yangmai 16) were sown into 40-L pots (45 cm in length, 30 cm in both width and height, with a valve near the bottom) filled with 16 kg deionized quartz sand. Eight seeds were sown in each pot. A Hoagland nutrient solution that contained 3.0 mM NH_4_NO_3_ (^15^NH_4_^15^NO_3_, 5.0% in ^15^N abundance), 1.0 mM KH_2_PO_4_, 2.0 mM KCl, 2.0 mM CaSO_4_, 1.0 mM MgSO_4_, 1.0 mM NaCl, 2.3 μM H_3_BO_3_, 0.46 μM MnSO_4_, 0.038 μM ZnSO_4_, 0.016 μM CuSO_4_, 0.006 μM H_2_MoO_4_, 89.3 μM FeSO_4_, and 43.1 μM Na_2_EDTA was used to provide nutrients to the plants. Depending on the winter wheat growth stages, four treatments were established: (1) NH_4_NO_3_ was replaced with ^15^${\text{NH}}_{4}^{15}$NO_3_ during the period of emergence (November 14^th^ 2011) to jointing (March 12^th^ 2012; EJ); (2) NH_4_NO_3_ was replaced with ^15^NH_4_^15^NO_3_ during the period of jointing-anthesis (April 12^th^ 2012; JA); (3) NH_4_NO_3_ was replaced with ^15^NH_4_^15^NO_3_ during the period of anthesis-maturity (2^nd^ June 2012; AM); and (4) NH_4_NO_3_ was applied during the whole growth season as the control (NN). The wheat plants were grown in a greenhouse to prevent any rainfall. Nine liters of nutrient solution with or without ^15^NH_4_^15^NO_3_ were applied to each pot every 7 days. The experiment was a completely randomized block design, with three replicates per treatment. Each replicate included six pots. Uniform tillers that flowered on the same day were tagged. All plants in one pot were harvested and separated into the root, stem, leaf, and spike (chaff and grain at maturity) for each replicate at anthesis and maturity. The samples were then dried at 80°C until a constant weight was reached. The pots left were harvested at maturity to record the grain yield.

Approximately 10 g of wheat grain was used for analysis of the whole grain N concentration and the concentrations of total protein and protein components. Following the methods of Liu et al. (2007), the wheat grain was pearled into eight fractions (P_1_–P_8_) from the surface layer to the center part, with two rice polishers (JNMJ7 and JNMJ6, Taizhou Grain Industry Instrument Corp, China). The pearling fractions were classified as P_1_ (0–10%), P_2_ (10–20%), P_3_ (20–30%), P_4_ (30–40%), P_5_ (40–50%), P_6_ (50–60%), P_7_ (60–70%), and P_8_ (70–80%). The pearling residue (20% of the whole grain) of each sample was ground in a stainless grinder (DJ-04B, Shanghai Dianjiu Machinery Manufactory, China) and designated as P_9_ (80–100%).

### Chemical analysis

The protein concentration in the whole wheat grain and the albumin, globulin, glutenin, and gliadin fractions were determined by the micro-Kjeldhl distillation method of AACC 46-13.01 with some modifications (Li et al., 2012).

The natural abundance and atom% of ^15^N in the plant samples were detected using a pattern ZHT-O2 mass spectrometer following the method by Shi et al. (2012b) at the Agroforestry Academy, Chemical Institute of Hebei Province, China.

### Calculation methods

The percentage of N that was derived from fertilizer-N (Ndff, %) was calculated by the following Equation (1) (Cookson et al., 2001; Shi et al., 2012b):

(1) $$\begin{array}{l} \text{Ndff}\left(\text{\%}\right)=\frac{c-b}{a-b}\times 100 \end{array}$$

where *a* is the atom% of ^15^N in the fertilizer, *b* is the atom% of ^15^N in the unfertilized plants, and *c* is the atom % of ^15^N in the fertilized plants.

The accumulation of N that was assimilated by the plants from fertilizer-N was calculated following Equations (2) and (3):

(2) $$\begin{array}{ll} & \text{Plant total N accumulation }\left(\text{mg}∕\text{plant}\right)\\ \;=\frac{\text{plant dry weight }\times \text{N concentration}}{100}\times 1000 \end{array}$$

(3) $$\begin{array}{ll} \; & \text{N from fertilizer N }\left(\text{mg}∕\text{plant}\right)=\frac{\left(2\right){\times \text{Ndff}}_{\text{plant}}}{100} \end{array}$$

The N distribution was calculated as the ratio of the ^15^N accumulation amount (mg) in a given organ to the total ^15^N accumulation in the whole plant (mg).

The remobilization of stored N from a given organ to grain (RAN) was calculated by subtracting the N at maturity from the N at anthesis in this organ. The translocation efficiency of the stored N in a given organ (TEN) was calculated following Equation (4):

(4) $$\begin{array}{l} \text{TEN}\left(\text{\%}\right)=\frac{\text{RAN}}{\text{N amonut at anthesis}}\times 100 \end{array}$$

The contribution of RAN to grain N (CRAN) was calculated following Equation (5):

(5) $$\begin{array}{l} \text{CRAN }\left(\text{\%}\right)=\;\frac{\text{RAN}}{\text{N amount in grain at maturity}}\times 100 \end{array}$$

### Statistical analysis

All of the data were subjected to one-way ANOVA using SPSS version 20.0 for windows (IBM SPSS Statistics, Chicago, IL, USA). The Duncan's multiple range test was used to check the significance of the difference between treatments.

## Results

### Distribution of assimilated N before anthesis in the organs at anthesis

**Figure 1:**
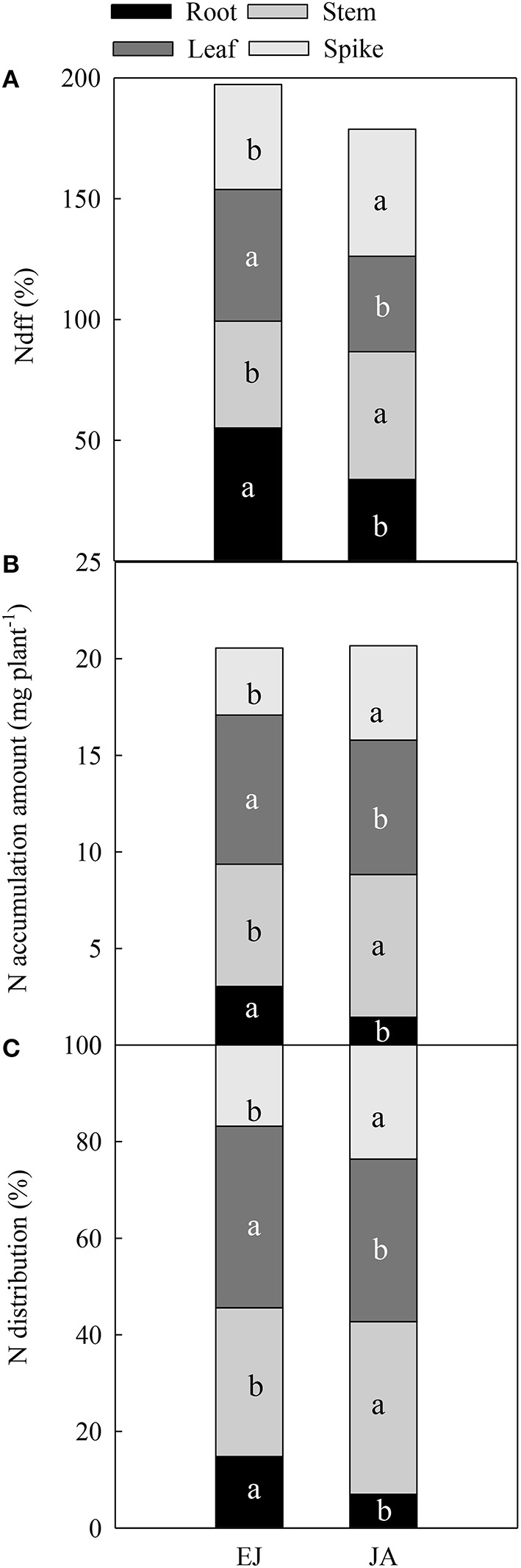
**Percentage of N derived from fertilizer-N (Ndff, %) (A), accumulation amount (B), and contribution of absorbed N (C) during periods from emergence to jointing (EJ), and from jointing to anthesis (JA)**. Different small letters for the same organ (root, stem, leaf, and spike) indicate significant difference at 0.05 level between different growth periods. Sheath was included in stem.

The percentages of N derived from fertilizer-N (Ndff, %) in all organs (root, stem, leaf, and spike) were different between during EJ (Emergence-Jointing) and JA (Jointing-Anthesis; Figure 1A). The values of Ndff in the root and leaf during EJ were significantly higher than those during JA, while an opposite trend was found in the Ndff in the stem and spike. The accumulation of N during EJ in the root and leaf was 11.1 and 10.9% higher than that during JA, while the N accumulation during EJ in the stem and spike was 14.3 and 29.1% lower than that during JA (Figure 1B). The distribution of N in the root and leaf that was absorbed during EJ was significantly higher than that during JA (*P* < 0.05; Figure 1C). On the contrary, the distribution of N in the stem and spike that was absorbed during EJ was significantly lower than that during JA.

### Remobilization of the stored N before anthesis to the grain

**Figure 2:**
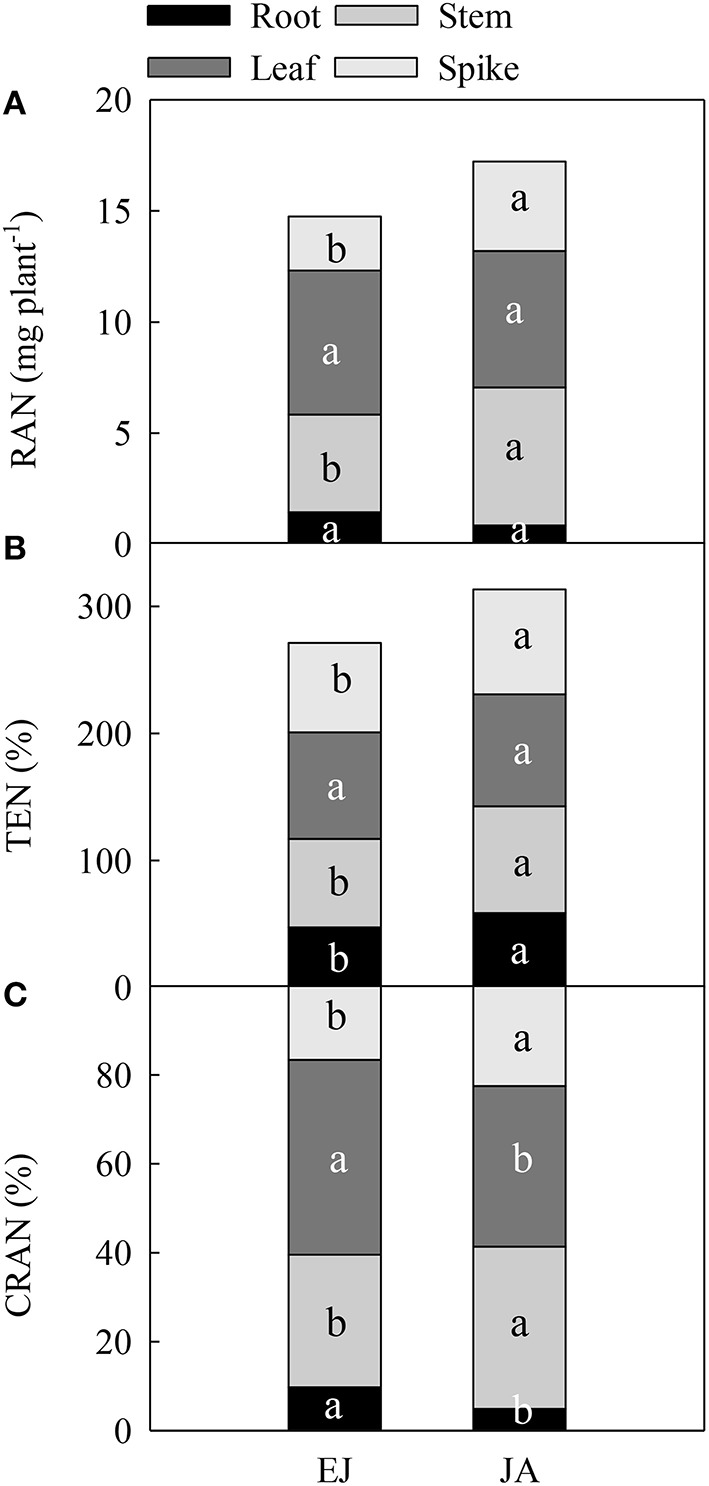
**Redistribution amount of N absorbed during periods from emergence to jointing (EJ) and from jointing to anthesis (JA) into grains (RAN) (A), translocation efficiency of the stored N in a given organ (TEN) (B), and contribution of RAN to grain N (CRAN) (C)**. Different small letters for the same organ (root, stem, leaf and spike) indicate significant difference at 0.05 level between different growth periods. Sheath was included in stem.

The remobilization of N (RAN) stored during EJ in the stem and spike to the grain was significantly lower than the RAN stored during JA (Figure 2A). However, for the leaf and root, no significant difference was found in RAN stored during EJ and JA. The translocation efficiency of N stored during EJ in the stem, spike and root (TEN) was significantly lower than TEN stored during JA (Figure 2B). The contribution of RAN (CRAN) stored in the leaf and root during EJ to grain was higher than the CRAN stored during JA (Figure 2C).

### Distribution of assimilated N at different periods in organs at maturity

**Figure 3:**
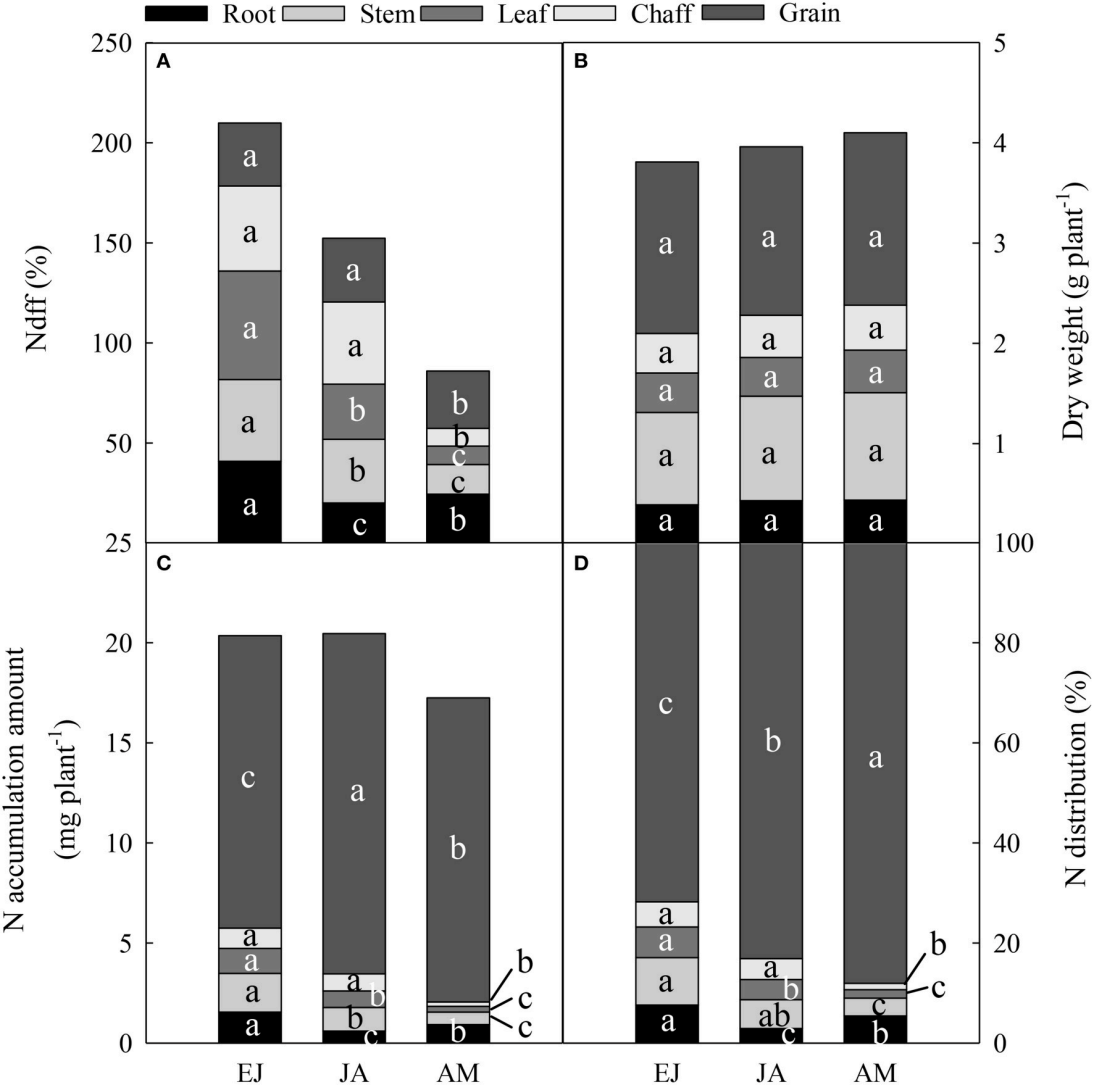
**Percentage of N derived from fertilizer-N (Ndff, %) (A), dry weight in different vegetative organs at maturity (B), accumulation amount (C), and contribution (D) of absorbed N during periods from emergence to jointing (EJ), from jointing to anthesis (JA) and from anthesis to maturity (AM)**. Different small letters for the same organ (root, stem, leaf, chaff, and grain) indicate significant difference at 0.05 level between different growth periods. The chaff includes spike axis and kernel husks. Sheath was included in stem.

The Ndff during EJ was the highest among all of the growth periods (Figure 3A). In the chaff and grain, the Ndff during EJ and JA was significantly higher than that during AM (*P* < 0.05). In the stem and leaf, the Ndff during JA was significantly higher than those during AM, but it was lower than that during EJ (*P* < 0.001). In the root, The Ndff during JA was significantly lower than that during AM (Anthesis-Maturity; *P* < 0.001).

There was no difference in dry weight of plants among these treatments at maturity (Figure 3B). The N accumulation in the chaff (including the spike axis and kernel husks) during EJ and JA were higher than that during AM (Figure 3C). The highest amount of N accumulation in the stem and leaf was found during EJ, followed by during JA, and the lowest was during AM. However, the highest N accumulation in the root was observed during EJ, followed by during AM, and the lowest was during JA. In addition, the distribution of N absorbed during AM was higher than that absorbed during EJ and JA (Figure 3D).

### Distribution of assimilated N at different periods in the pearling fractions of the grain

Across the pearling fractions in the grain, the highest Ndff during EJ was found in the P_2_ fraction, whereas the lowest Ndff was in the fractions P_6_ and P_9_ (Table 1). The Ndff during JA showed a decreasing trend from the surface layer to the center part, where the highest Ndff were found in the fractions P_1_, P_2_, and P_3_. However, the Ndff during AM showed an opposite trend: the highest Ndff-values were in P_7_ and P_8_. The highest accumulation of N was found in the P_9_ fraction in all these treatments. In addition, the average amount of N accumulation in the P_1_–P_4_ fractions was higher than in the P_5_–P_9_ fractions. In each pearling fraction the N was mostly accumulated during JA. The distribution of N assimilated during EJ, JA, and AM increased from the surface layer to the center part (except for P_1_ and P_9_).

**Table 1:** **Percentage of ^15^N derived from fertilizer-N (Ndff, %), accumulation and distribution of absorbed N during different growing periods in different pearling fractions of wheat grain**.

**Pearling fractions**	**Ndff (%)**			**N accumulation amount (mg plant^−1^)**			**N contribution (%)**			**N distribution (%)**		
	**EJ**	**JA**	**AM**	**EJ**	**JA**	**AM**	**EJ**	**JA**	**AM**	**EJ**	**JA**	**AM**
P_1_	31.14c	31.59a	25.10f	1.82b	2.16c	1.67d	8.87b	10.43c	9.66d	12.35c	12.67c	11.68d
P_2_	31.70a	31.70a	26.65e	2.10a	2.31b	1.86b	10.23a	11.16b	10.81b	14.25b	13.56b	13.06b
P_3_	31.40b	31.58a	27.08d	1.84b	2.13c	1.76c	8.97b	10.30c	10.19c	12.50c	12.51c	12.31c
P_4_	31.05c	31.31b	27.36c	1.62c	1.83d	1.54e	7.89c	8.84d	8.92e	10.98d	10.74d	10.78e
P_5_	31.00c	31.37b	27.80b	1.46d	1.69e	1.45f	7.13d	8.19e	8.43f	9.93e	9.95e	10.19f
P_6_	30.23e	31.01c	27.87b	1.43d	1.62e	1.41f	6.96d	7.85e	8.17f	9.70e	9.53e	9.87f
P_7_	30.40d	31.04c	28.32a	1.20e	1.42f	1.23g	5.83e	6.85f	7.15g	8.12f	8.32f	8.64g
P_8_	30.38de	31.09c	28.28a	1.10f	1.25g	1.14h	5.34f	6.03g	6.62h	7.43g	7.32g	7.99h
P_9_	30.23e	31.10c	27.27c	2.18a	2.62a	2.21a	10.59a	12.68a	12.81a	14.74a	15.40a	15.48a
P_1−4_	31.32	31.55	26.55	1.85	2.11	1.71	8.99	10.18	9.90	12.52	12.37	11.96
P_5−9_	30.45	31.12	27.91	1.43	1.24	5.98	7.20	8.32	8.42	30.45	31.12	27.91
P_5−9_/P_1−4_	0.97	0.99	1.05	0.68	0.73	0.66	0.73	0.66	0.68	0.97	0.99	1.05

P_1_–P_9_, pearling fraction 1–9; P_1–4_, mean value of P_1_–P_4_; P_5–9_, mean value of P_5_–P_9_; P_5–9_/P_1–4_, ratio of P_5–9_ to P_1–4_. EJ, emergence-jointing; JA, jointing-anthesis; AM, anthesis-maturity. The small letters in the same column indicate significant difference at 0.05 level among different pearling fractions under given treatment. The mean value of N accumulation amount, N contribution and N distribution in fractions of P_5–9_ were the total values divided by 6, respectively, since the P_5–9_ fractions accounted for 60% of the total grain mass.

### Distribution of the assimilated N in the protein fractions of pearled grains

**Figure 4:**
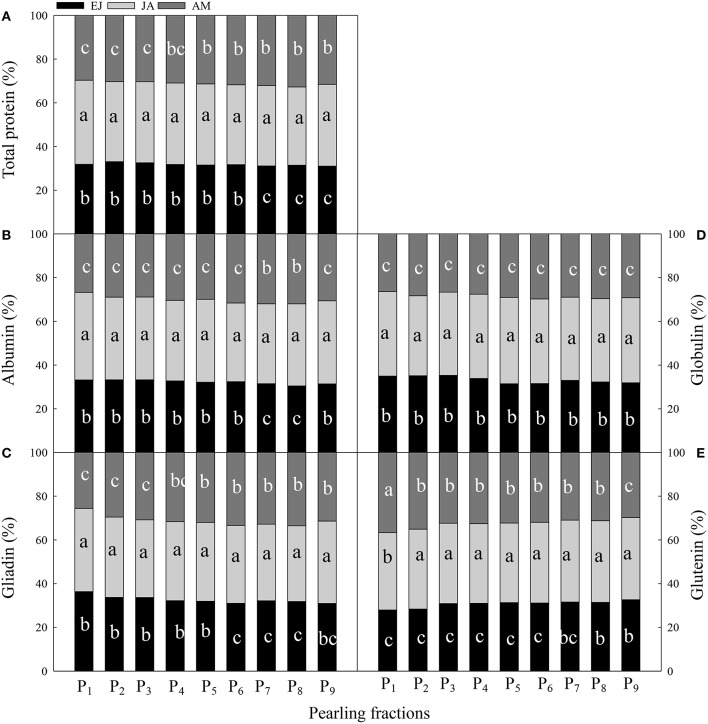
**Contribution of absorbed N druing periods from emergence to jointing (EJ), from jointing to anthesis (JA) and from anthesis to maturity (AM) to the total protein (A) and protein components (albumin, B; globulin, C; gliadin, D; glutenin, E) of different pearling fractions of wheat grain**. P1–P9, pearling fraction 1–9. Different small letters for the same period (EJ, from emergence stage to jointing stage; JA, from jointing to anthesis stage; AM, from anthesis to maturity stage) indicate significant difference at 0.05 level between pearling fractions.

The contribution of assimilated N during JA to the protein and protein fractions in each layer was the highest, except for the glutenin concentration in the P_1_ fraction (Figure 4). For the total protein, the contribution of N assimilated during EJ was higher than that during AM in the fractions P_1_–P_6_; however, it was opposite in the fractions P_7_–P_9_. For albumin, the contribution of N assimilated during EJ was significantly higher than during AM in the fractions P_1_–P_6_ and P_9_, and the contribution of N assimilated during EJ to the globulin was significantly higher than that during AM in all of the fractions. For gliadin, the N assimilated during EJ contributed more than that during AM in the fractions P_1_–P_3_, whereas the opposite was true in the fractions P_4_–P_9_. In the fractions of P_2_–P_8_, N assimilated during AM contributed more to glutenin than that during EJ; however, in fraction P_1_, the N contribution was found to be in the order: during AM> during JA> during EJ.

**Figure 5:**
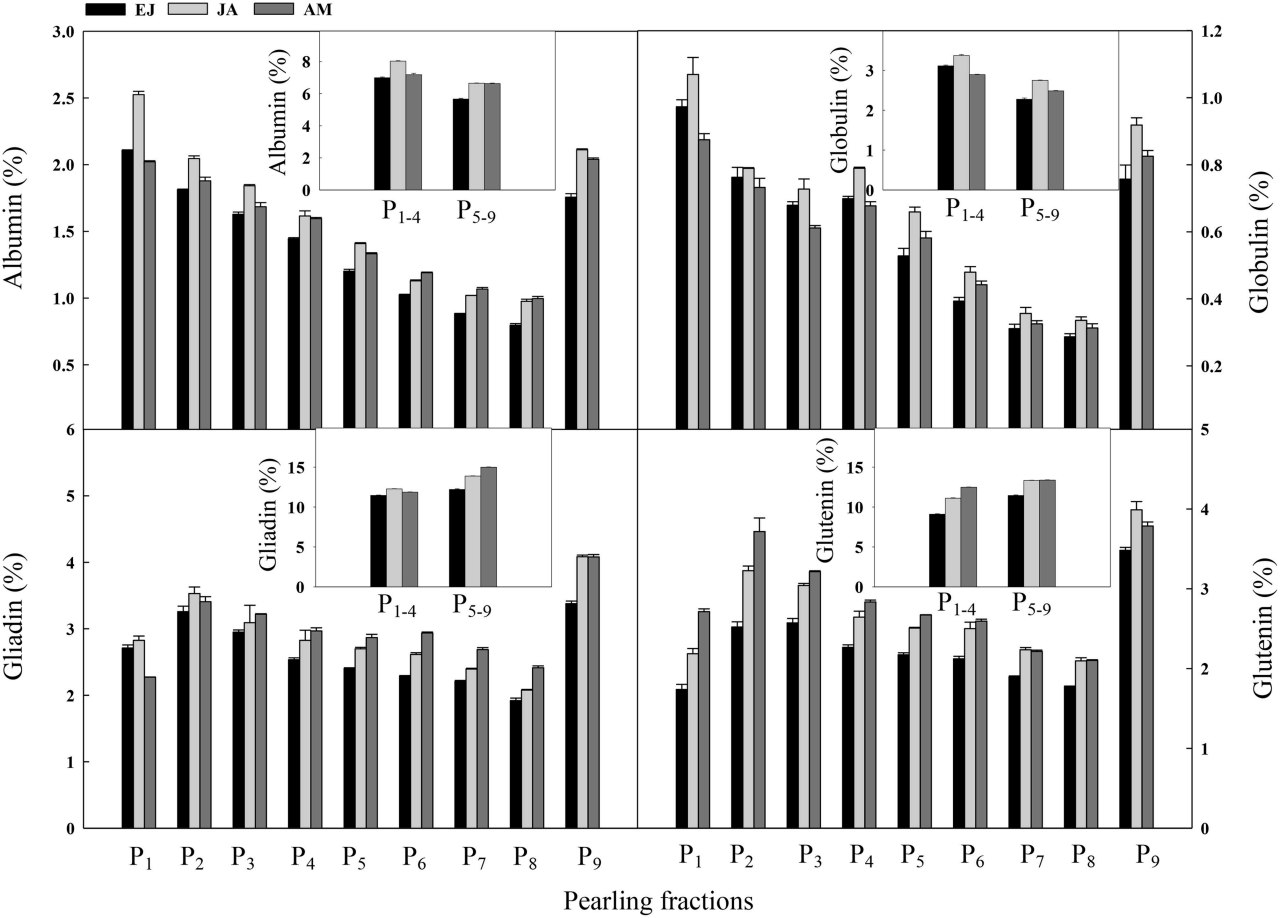
**Distribution of absorbed N druing periods of emergence-jointing (EJ), jointing-anthesis (JA), and anthesis-maturity (AM) in the protein components of different pearling fractions of wheat grain**. P_1_–P_9_, pearling fraction 1-9; P_1–4_, mean value of P_1_–P_4_; P_5–9_, mean value of P_5_–P_9_.

Across the pearling fractions, the distribution of N assimilated during EJ, JA, and AM in albumin and globulin was decreased from the surface layer to the center part (Figure 5). In albumin, the distribution of N assimilated during JA was higher than that during the other two periods in P_1−4_. Also, a decreased trend from P_2_ to P_8_ was found in both gliadin and glutenin. For gliadin, the distribution of N assimilated during JA was higher than those during the other two periods in P_1−4_, and in P_5−9_ the distribution of N assimilated during AM was the highest among these three periods. For glutenin, in P_1−4_ the distribution of N assimilated during AM was the highest among the three periods, and in P_5−9_ the distribution of N assimilated during JA and AM was higher than that during EJ.

## Discussion

### Distribution and remobilization of N stored at different periods

The N utilization of plants involves several processes, such as uptake, assimilation, translocation, and remobilization (Masclaux-Daubresse et al., 2008). Our study illustrated the spatial and temporal N distribution in the grains, using ^15^N isotope tracing technique. During vegetative growth stages, the leaves have the highest N accumulation of any component because the photosynthetic apparatus is the greatest sink of N in cereal plants (Flood and Martin, 2001). In this study, the highest N accumulation was found in the leaves at anthesis, which mostly accumulated before jointing (Figure 1). However, the N that accumulated in the stem during JA was higher than that accumulated during EJ because of the rapid stem extension after jointing (Figure 1). During the initial phase of the grain growth, N accumulation continues in the stem and leaf, while the grain turn to be the major sink of N (Ferrise et al., 2010). In agreement with the study of Bertheloot et al. (2008) with bread wheat, here the N concentration in all of the organs started to decrease after anthesis with a rate that was proportional to their N concentration (Figure 2). The leaf and stem have nearly equal contributions to the grain N, but the main part of remobilized N, e.g., amino acids (lysine and proline) and small peptides, from these two organs was accumulated at different stages. For example, in leaf the remobilization of N stored during EJ was higher than the N stored during JA, whereas in the stems the remobilization of N stored during JA was higher than the N stored during EJ.

Rapid accumulation of N in grain after anthesis resulted in more than 70% of the total N being in the grains at maturity, which was mainly attributed to the remobilization of N assimilated during JA (Figure 3). In addition, the main part of the N was stored temporarily in the stems among all of the vegetative tissues after anthesis (Figure 3). Simpson et al. (1983) reported that the leaves, glumes, stems, and roots contribute 40, 23, 23, and 16%, respectively, to the daily rate of grain N accumulation in wheat at mid-grain filling. Here, the contribution of N stored during EJ was 43.8, 29.8, 9.5, and 16.5% from the leaves, stem, roots, and spike, respectively, to the grain. The N stored during JA in the leaves, stem, roots and spike contributed 36.1, 36.5, 5.0, and 23.7%, respectively, to the grain (Figure 2). This finding indicates that the contribution of N stored in the stems to the grain N increased during later vegetative growth stages before anthesis.

The Ndff during EJ was significantly higher than that during JA and AM in all of the organs, except for in the chaff and grain (Figure 3), suggesting that N assimilated during EJ is significantly involved in the functioning of meristematic tissue. It has been reported that up to 75% of the reduced N in cereal leaves is located in mesophyll cells, mostly as Rubisco (Bertheloot et al., 2008). On the other hand, the N absorbed and stored during JA and AM was mainly involved in the N metabolism as mobile constituents, which is remobilized to the growing grain during the reproductive stage (Bertheloot et al., 2008). Consistent with this, here higher accumulations and distributions of N stored during JA and AM than during EJ in mature grain were found.

### Distribution of N stored at different periods in the protein fractions of pearled grains

Wheat grain consists of the embryo, starchy endosperm, and bran. Among these three distinct parts, the bran comprises the pericarp (fruit coat), testa (seed coat), and outer endosperm (aleurone layer), which was included in fraction P_1_ in the present study (Mousia et al., 2004). The starchy endosperm consists of an outer aleurone layer and inner columns of starchy endosperm cells, which are packed with most of the starch and protein, accounting for approximately 80% of the grain mass (Tosi et al., 2011). During milling, the germ and bran are removed, leaving the starchy endosperm as the principal contributor to white flour (Dupont and Altenbach, 2003). Typically, the protein concentration is relatively low in the cells near the endosperm cavity and increases in an outward radial direction in a mature endosperm (Farrand and Hinton, 1974; Tosi et al., 2011). In the present study, the N accumulation also showed an increased trend from P_8_ to P_2_, where the endosperm is located (Table 1). Although it has been well-known that the gradients in the protein distribution are present in cereal grains, the underlying mechanisms by which the gradient was established during grain filling is still unclear. Tosi et al. (2011) hypothesized that the gradients have a developmental basis because both the quantitative and qualitative gradients of the protein distribution appear to follow the radial pattern of cell development in the endosperm. They concluded that the gradients in the gluten protein composition are related to the origin of the subaleurone cells, which are different from other starchy endosperm cells that derived from the re-differentiation of aleurone cells, but it could also be related to the signals produced by the maternal tissue that affect specific domains of the gluten protein gene promoters (Tosi et al., 2011).

It should be noted that the Ndff during EJ and JA increased from the inner starchy endosperm to the outer layer with a peak value in P_2_, while it was decreased in P_1_ (which corresponds to the outer layers, aleurone, and subaleurone; Table 1). However, the Ndff during AM decreased from P_8_ (inner layer) to P_1_. The varied trends in Ndff during the different growing stages could be explained by the fact that two separate pathways may operate in the developing wheat grain (Tosi et al., 2009). It could be hypothesized that the remobilization of N that stored before anthesis depends on the apoplast transport through the aleurone layer to the endosperm cells, and the N assimilated after anthesis is transferred following the radial direction in the endosperm cells. Nevertheless, the establishment of these gradients should be further studied considering the biological process of the grain development.

The contributions of N assimilated during different periods to the grain protein fractions varied across all of the layers, although the largest contribution was found from N assimilated during JA in each layer (Figure 4). For the albumin and gliadin, the contribution of N assimilated during EJ and JA declined from the outer layer to the inner layer, whereas the N contribution assimilated after anthesis showed a reversed trend. For globulin, the contribution of N assimilated during EJ decreased, whereas that during JA and AM increased from the outer layer to the inner layer. For glutenin, the contribution of N assimilated before anthesis increased, whereas the N contribution assimilated after anthesis decreased from the outer layer to the inner layer. The different contributions of N after anthesis to the protein fractions could be related to the synthesis process and transport pathway of each protein fraction during grain development (Zhu et al., 2005). The main grain structural proteins are synthesized during early grain growth, and later, the storage proteins, including gliadin and glutenin, are accumulated (Stone and Nicolas, 1996). The gliadin is a compound accumulated relatively late but fast, and part of the structural proteins can be converted into storage proteins during the latter grain-filling stage (Stone and Nicolas, 1996).

The distributions of N in grain assimilated during different periods across all of the layers showed different trends (Figure 5). For example, for albumin and gliadin, the N assimilated during JA had a high distribution ratio in the outer layer, whereas the contribution of N assimilated after anthesis increased faster than that before anthesis. This finding indicates that the utilization efficiency of N assimilated during JA is higher for structural proteins and greater amount of structural proteins were converted to storage proteins in the inner layers. However, for globulin and glutenin, the N assimilated after anthesis had the highest distribution ratio in most of the layers indicating that the utilization efficiency of those N is higher than that assimilated before anthesis for storage proteins, especially in the inner layers. This result is in accordance with the finding that the increased N application rate after anthesis can enhance the protein concentration in the endosperm of wheat grain (Johansson et al., 2004).

In conclusion, our results indicated that the remobilization of N stored from jointing to anthesis was the major contributor to the grain N, especially in the outer layer. The distributions of N assimilated during EJ, JA, and AM in albumin and globulin showed a decreased trend from the surface layer to the center part. For globulin and glutenin, the N stored after anthesis had the highest distribution ratio in most of the layers, which indicates that the utilization efficiency of N assimilated after anthesis is higher than that before anthesis for the storage proteins. Therefore, modification in the N application rate during the different growth stages should be an effective approach to regulating the distribution of the protein fractions in the pearled grains for specific end-use. However, the establishment of these gradients should be further studied while considering the biological processes that are involved in the grain development.

## Author contributions

DJ and QZ designed research; XL and LZ performed the experiment; XW, JC, XL, and LZ analyzed the data; LF, TD, WC, and DJ revised the manuscript.

### Conflict of interest statement

The authors declare that the research was conducted in the absence of any commercial or financial relationships that could be construed as a potential conflict of interest.
